# Supplementary material for: COZOID: contact zone identifier for visual analysis of protein-protein interactions
Source: BMC Bioinformatics. 2018 Apr 6;19:125. doi: 10.1186/s12859-018-2113-6 (PMC5889581; doi:10.1186/s12859-018-2113-6)
Supplement: Supplementary file 2 — Software build. Executable binary file of the software tool. (ZIP 118,784 kb) [file 12859_2018_2113_MOESM2_ESM.zip › cozoid/java/docs/beansbinding-1.2.1-doc.zip!/index-files/index-2.html]

B-Index (Beans Binding)


---


|  |  |  |
| --- | --- | --- |
| **Overview** \| Package \| Class \| Use \| **Tree** \| **Deprecated** \| **Index** \| **Help** | |  |
| **PREV LETTER**   **NEXT LETTER** | **FRAMES**    **NO FRAMES**     **All Classes** |  |


A B C D E F G H I J K L M N O P Q R S T U V W 

---


## **B**

**backup(int)** - Method in class org.jdesktop.el.impl.parser.SimpleCharStream: **BADLY\_ESCAPED\_STRING\_LITERAL** - Static variable in interface org.jdesktop.el.impl.parser.ELParserConstants: **base** - Variable in class org.jdesktop.el.impl.parser.AstValue.Target: **BeanAdapterBase** - Class in org.jdesktop.swingbinding.adapters: **BeanAdapterBase(String)** - Constructor for class org.jdesktop.swingbinding.adapters.BeanAdapterBase: **BeanAdapterFactory** - Class in org.jdesktop.beansbinding.ext: **BeanAdapterFactory()** - Constructor for class org.jdesktop.beansbinding.ext.BeanAdapterFactory: **BeanAdapterProvider** - Interface in org.jdesktop.beansbinding.ext: **BeanELResolver** - Class in org.jdesktop.el: Defines property resolution behavior on objects using the JavaBeans component architecture. **BeanELResolver()** - Constructor for class org.jdesktop.el.BeanELResolver: Creates a new read/write `BeanELResolver`. **BeanELResolver(boolean)** - Constructor for class org.jdesktop.el.BeanELResolver: Creates a new `BeanELResolver` whose read-only status is determined by the given parameter. **BeanELResolver.BeanProperties** - Class in org.jdesktop.el: **BeanELResolver.BeanProperties(Class<?>)** - Constructor for class org.jdesktop.el.BeanELResolver.BeanProperties: **BeanELResolver.BeanProperty** - Class in org.jdesktop.el: **BeanELResolver.BeanProperty(Class<?>, PropertyDescriptor)** - Constructor for class org.jdesktop.el.BeanELResolver.BeanProperty: **BeanProperty**<S,V> - Class in org.jdesktop.beansbinding: An implementation of `Property` that uses a simple dot-separated path syntax to address Java Beans properties of source objects. **beginColumn** - Variable in class org.jdesktop.el.impl.parser.Token: beginLine and beginColumn describe the position of the first character of this token; endLine and endColumn describe the position of the last character of this token. **beginLine** - Variable in class org.jdesktop.el.impl.parser.Token: beginLine and beginColumn describe the position of the first character of this token; endLine and endColumn describe the position of the last character of this token. **BeginToken()** - Method in class org.jdesktop.el.impl.parser.SimpleCharStream: **BIGDECIMAL** - Static variable in class org.jdesktop.el.impl.lang.ELArithmetic: **BIGINTEGER** - Static variable in class org.jdesktop.el.impl.lang.ELArithmetic: **bind()** - Method in class org.jdesktop.beansbinding.Binding: Binds this binding. **bind()** - Method in class org.jdesktop.beansbinding.BindingGroup: Calls `bind` on all unbound bindings in the group. **bindImpl()** - Method in class org.jdesktop.beansbinding.AutoBinding: **bindImpl()** - Method in class org.jdesktop.beansbinding.Binding: Called by `Binding.bind()` to allow subclasses to initiate binding. **bindImpl()** - Method in class org.jdesktop.swingbinding.impl.AbstractColumnBinding: **bindImpl()** - Method in class org.jdesktop.swingbinding.JComboBoxBinding: **bindImpl()** - Method in class org.jdesktop.swingbinding.JListBinding: **bindImpl()** - Method in class org.jdesktop.swingbinding.JTableBinding: **Binding**<SS,SV,TS,TV> - Class in org.jdesktop.beansbinding: `Binding` is an abstract class that represents the concept of a binding between two properties, typically of two objects, and contains methods for explicitly syncing the values of the two properties. **Binding(SS, Property<SS, SV>, TS, Property<TS, TV>, String)** - Constructor for class org.jdesktop.beansbinding.Binding: Create an instance of `Binding` between two properties of two objects. **Binding.SyncFailure** - Class in org.jdesktop.beansbinding: `SyncFailure` represents a failure to sync (`save` or `refresh`) a `Binding`. **Binding.SyncFailureType** - Enum in org.jdesktop.beansbinding: An enumeration representing the reasons a sync (`save` or `refresh`) can fail on a `Binding`. **Binding.ValueResult**<V> - Class in org.jdesktop.beansbinding: Encapsulates the result from calling `Binding.getSourceValueForTarget()` or `Binding.getTargetValueForSource()`, which can either be a successful value or a failure. **bindingBecameBound(Binding)** - Method in class org.jdesktop.beansbinding.AbstractBindingListener: Notification that a `Binding` has been bound. **bindingBecameBound(Binding)** - Method in interface org.jdesktop.beansbinding.BindingListener: Notification that a `Binding` has been bound. **bindingBecameUnbound(Binding)** - Method in class org.jdesktop.beansbinding.AbstractBindingListener: Notification that a `Binding` has been unbound. **bindingBecameUnbound(Binding)** - Method in interface org.jdesktop.beansbinding.BindingListener: Notification that a `Binding` has been unbound. **BindingGroup** - Class in org.jdesktop.beansbinding: `BindingGroup` allows you to create a group of `Bindings` and operate on and/or track state changes to the `Bindings` as a group. **BindingGroup()** - Constructor for class org.jdesktop.beansbinding.BindingGroup: Creates an empty `BindingGroup`. **BindingListener** - Interface in org.jdesktop.beansbinding: `BindingListeners` are registered on `Bindings` or `BindingGroups` to listen for changes to the state of `Bindings` **Bindings** - Class in org.jdesktop.beansbinding: A factory class for creating instances of the concrete `Binding` implementations provided by this package. **bindUnmanaged()** - Method in class org.jdesktop.beansbinding.Binding: A protected version of `Binding.bind()` that allows managed subclasses to bind without throwing an exception for being managed. **Boolean()** - Method in class org.jdesktop.el.impl.parser.ELParser: **BooleanNode** - Class in org.jdesktop.el.impl.parser: **BooleanNode(int)** - Constructor for class org.jdesktop.el.impl.parser.BooleanNode: **BracketSuffix()** - Method in class org.jdesktop.el.impl.parser.ELParser: **bufcolumn** - Variable in class org.jdesktop.el.impl.parser.SimpleCharStream: **buffer** - Variable in class org.jdesktop.el.impl.parser.SimpleCharStream: **bufline** - Variable in class org.jdesktop.el.impl.parser.SimpleCharStream: **bufpos** - Variable in class org.jdesktop.el.impl.parser.SimpleCharStream: **bundle** - Static variable in class org.jdesktop.el.impl.util.MessageFactory

---


|  |  |  |
| --- | --- | --- |
| **Overview** \| Package \| Class \| Use \| **Tree** \| **Deprecated** \| **Index** \| **Help** | |  |
| **PREV LETTER**   **NEXT LETTER** | **FRAMES**    **NO FRAMES**     **All Classes** |  |


A B C D E F G H I J K L M N O P Q R S T U V W 

---
